# Breakthroughs in Medicinal Chemistry: New Targets and Mechanisms, New Drugs, New Hopes-3

**DOI:** 10.3390/molecules23071596

**Published:** 2018-06-30

**Authors:** Arduino A. Mangoni, Tiziano Tuccinardi, Simona Collina, Jean Jacques Vanden Eynde, Diego Muñoz-Torrero, Rafik Karaman, Carlo Siciliano, Maria Emília de Sousa, Katalin Prokai-Tatrai, Jarkko Rautio, Catherine Guillou, Michael Gütschow, Stefania Galdiero, Hong Liu, Luigi A. Agrofoglio, Jean-Marc Sabatier, Christopher Hulme, George Kokotos, Qidong You, Paula A. C. Gomes

**Affiliations:** 1 Department of Clinical Pharmacology, Flinders University and Flinders Medical Centre, Bedford Park, SA 5042, Australia; arduino.mangoni@flinders.edu.au; 2 Department of Pharmacy, University of Pisa, Via Bonanno 6, 56126 Pisa, Italy; tiziano.tuccinardi@unipi.it; 3 Department of Drug Sciences, Medicinal Chemistry and Pharmaceutical Technology Section, Centre for Health Technologies (CHT), University of Pavia, Viale Taramelli 12, 27100 Pavia, Italy; simona.collina@unipv.it; 4 Formerly head of the Department of Organic Chemistry (FS), University of Mons-UMONS, 7000 Mons, Belgium; jean-jacques.vandeneynde@ex.umons.ac.be; 5 Laboratory of Pharmaceutical Chemistry, Faculty of Pharmacy and Food Sciences, and Institute of Biomedicine (IBUB), University of Barcelona, Av. Joan XXIII, 27-31, Barcelona E-08028, Spain; 6 Pharmaceutical & Medicinal Chemistry Department, Faculty of Pharmacy, Al-Quds University, POB 20002 Jerusalem, Palestine; dr_karaman@yahoo.com; 7 Department of Sciences, University of Basilicata, Viadell’Ateneo Lucano 10, 85100 Potenza, Italy; 8 Department of Pharmacy, Health and Nutritional Sciences, University of Calabria, I-87036 Arcavacata di Rende, Italy; carlo.siciliano@unical.it; 9 Laboratório de Química Orgânica e Farmacêutica, Departamento de Ciências, Químicas, Faculdade de Farmácia, Universidade do Porto, Rua Jorge Viterbo Ferreira 228, 4050-313 Porto, Portugal; esousa@ff.up.pt; 10 Interdisciplinar de Investigação Marinha e Ambiental (CIIMAR/CIMAR), Universidade do Porto, Terminal de Cruzeiros do Porto de Leixões, Avenida General Norton de Matos, S/N 4450-208 Matosinhos, Portugal; 11 Department of Pharmacology and Neuroscience, and the Institute for Healthy Aging, University of North Texas Health Science Center, 3500 Camp Bowie Boulevard, Fort Worth, TX 76107, USA; Katalin.Prokai@unthsc.edu; 12 School of Pharmacy, Faculty of Health Sciences, University of Eastern Finland, P.O. Box 1627, FI-70211 Kuopio, Finland; jarkko.t.rautio@uef.fi; 13 Institut de Chimie des Substances Naturelles, CNRS UPR 2301, Université de Paris-Saclay, 91198 Gif-sur-Yvette, France; catherine.guillou@cnrs.fr; 14 Pharmaceutical Institute, University of Bonn, An der Immenburg 4, 53115 Bonn, Germany; guetschow@uni-bonn.de; 15 Department of Pharmacy, CIRPEB-University of Naples “Federico II”, Via Mezzocannone 16, 80134 Napoli, Italy; sgaldier@unina.it; 16 Key Laboratory of Receptor Research, Shanghai Institute of Materia Medica, Chinese Academy of Sciences, 555 Zu Chong Zhi Road, Shanghai 201203, China; hliu@simm.ac.cn; 17 ICOA UMR CNRS 6005, Universite d'Orleans, Rue de Chartres, 45067 Orleans CEDEX 2, France; luigi.agrofoglio@univ-orleans.fr; 18 Laboratory INSERM UMR 1097, Aix-Marseille University, 163, Parc Scientifique et Technologique de Luminy, Avenue de Luminy, Bâtiment TPR2, Case 939, Marseille 13288, France; sabatier.jm1@libertysurf.fr; 19 Department of Pharmacology and Toxicology, and Department of Chemistry and Biochemistry, College of Pharmacy, The University of Arizona, Biological Sciences West Room 351, 1041 East Lowell Street, Tucson, AZ 85721, USA; hulme@pharmacy.arizona.edu; 20 Laboratory of Organic Chemistry, Department of Chemistry, National and Kapodistrian University of Athens, Panepistimiopolis, Athens 15771, Greece; gkokotos@chem.uoa.gr; 21 State Key Laboratory of Natural Medicines and Jiangsu Key Laboratory of Drug Design and Optimization, China Pharmaceutical University, Nanjing, 210009, China; youqd@cpu.edu.cn; 22 LAQV-REQUIMTE, Departamento de Química e Bioquímica, Faculdade de Ciências da Universidade do Porto, Rua do Campo Alegre 687, 4169-007 Porto, Portugal; pgomes@fc.up.pt

## 1. Introduction

*Breakthroughs in Medicinal Chemistry: New Targets and Mechanisms, New Drugs, New Hopes* is a series of *Editorials*, which is published on a biannual basis by the Editorial Board of the Medicinal Chemistry section of the journal *Molecules*. In these *Editorials*, we highlight in brief reports (of about one hundred words) a number of recently published articles that describe crucial findings, such as the discovery of novel drug targets and mechanisms of action, or novel classes of drugs, which may inspire future medicinal chemistry endeavors devoted to addressing prime unmet medical needs.

## 2. A Hydrogen Peroxide Sensitive Prodrug Strategy for the Targeted Delivery of Methotrexate in Rheumatoid Arthritis 

Highlighted by Arduino A. Mangoni

Systemic exposure to methotrexate, an anti-inflammatory and immunomodulating drug, is associated with haematological, gastrointestinal, and lung toxicity. A targeted drug delivery in tissues affected by inflammation might retain therapeutic efficacy whilst preventing exposure to normal organs and tissues. Peiró Cadahía et al. investigated a methotrexate prodrug strategy consisting of a reactive oxygen species-labile 4-methylphenylboronic acid linked to methotrexate via a carbamate linkage, that led to the synthesis of a novel prodrug, (4-(((2-amino-4-((((4-boronobenzyl)oxy)carbonyl)amino)pteridin-6-yl)methyl)(methyl)amino)benzoyl)-l-glutamic acid [1]. Incubation of this prodrug with reactive oxygen species, at concentrations similar to those observed in inflammatory tissues, led to the exclusive release of methotrexate. In an animal model of inflammatory arthritis, this prodrug significantly reduced the macroscopic arthritis score when compared to the control group. The effect of the prodrug on the arthritis score was similar to that observed with methotrexate. Notably, progressive weight loss, a marker of toxicity, was observed with methotrexate, but not with the prodrug. A hydrogen peroxide sensitive prodrug strategy for the targeted delivery of methotrexate is a novel, promising, approach for the treatment of patients with rheumatoid arthritis.

## 3. A Novel Modulator of Rod Opsin Showing in Vivo Efficacy for Retinal Degeneration

Highlighted by Tiziano Tuccinardi

Mutations destabilizing rod opsin are one of the main factors that lead to retinal degeneration. Focusing on the analysis on the P23H rod opsin mutation, that is the most common mutation among autosomal dominant retinis pigmentosa patients in North America, Palczewski and co-workers reported compound YC-001 as the first non-retinal modulator of rod opsin with both inverse and non-competitive antagonist activities [2]. YC-001 was discovered by a cellular high-throughput screening; it showed an interesting activity in various *in vitro* binding assays, and showed *in vivo* efficacy in a mouse model of bright light-induced retinal degeneration, thus supporting the possible therapeutic application of this compound. The authors were not able to obtain a crystal structure of the YC-001:opsin complex; however, this study paves the way for the design and development of new candidate drugs for the treatment of pathologies associated with retinal degeneration.

## 4. Combining Innovative 2D NMR Techniques and Deep Neural Networks to Assist Natural Products Discovery

Highlighted by Simona Collina

In Nature-Aided-Drug-Discovery process, compound structure determination and extract dereplication are crucial and extremely time-consuming steps. To speed up the research in the field of natural compounds, the interdisciplinary team coordinated by Cottrell and Gerwick recently developed a tool called the *Small Molecule Accurate Recognition Technology* (SMART) [3]. The SMART tool uses a piece of spectral data which is unique and characteristic to each molecule, and runs it through a deep learning neural network to place the unknown molecule in a cluster of molecules with similar structures. In more detail, the HSQC-NMR of each compound produces a topological map of spots, and the Convolutional Neural Network (CNN) learning system takes the images of spectra of unknown molecules and maps them into a ten-dimensional space near molecules with similar traits. As the Authors stated, it is possible to associate and integrate biological, pharmacological, and ecological data with SMART, and thereby create new tools for enhanced discovery and development of biologically active natural products. Of note, SMART is not a “closed system”, and the continuous, further addition of compounds to the training set will improve accuracy and robustness of the system, thus accelerating natural product structural elucidation. 

## 5. Sertraline, Paroxetine, and Chlorpromazine are Rapidly Acting Anthelmintic Drugs Capable of Clinical Repurposing

Highlighted by Jean Jacques Vanden Eynde

**Figure 1:**
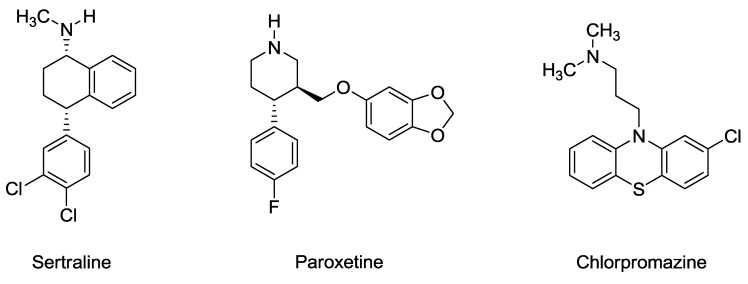
The structures of sertraline, paroxetine, and chlorpromazine.

Accelerating the identification of promising drug candidates and decreasing the costs associated with getting them to market are among the most important goals for the pharmaceutical industry [4]. A recent publication by J.C. Weeks et al. [5] has opened new doors in the search for innovative treatments of helminth (worm) infections. The authors performed a high-throughput screening of 218 compounds from the NIH Clinical Collection aimed at identifying hits that can perturb the life cycle of *Caenorhabditis elegans*, a nematode worm often used as a model for many human diseases. Three common drugs emerged: sertraline, paroxetine, and chlorpromazine (Figure 1). The first two are prescribed as antidepressants, whereas chlorpromazine is known as an antipsychotic. The study discovered that the three drugs act on *C. elegans* through non-canonical modes of action, and also measured their broad spectrum anti-parasitic effects against the parasitic nematodes, *Trichuris* and *Ancylostoma,* and the *Schistosoma* flatworm. The authors concluded that repurposing these commercially available medications, in combination with conventional anthelmintic drugs, could improve efficacy and limit the emergence of drug-resistant helminths. 

## 6. Turning a Gram-Positive-Only into an Effective Gram-Negative Antibiotic Using the Bacterial Machinery

Highlighted by Diego Muñoz-Torrero

Poor permeation through the outer membrane and degradation by β-lactamases are behind the lack of activity of many antibiotics against Gram-negative bacteria, some of which are among the most threatening pathogens for human health. Liu et al. have developed a very elegant approach to killing Gram-negative bacteria, using a Gram-positive oxazolidinone antibiotic conjugated with a cephalosporin and a bis-catechol-based siderophore [6]. The three-component conjugate displays a very original mechanism of action, which takes advantage of (i) bacterial ferri-siderophore uptake transporters to allow for the efficient passage of the conjugate through the outer membrane, and (ii) the periplasmic β-lactamases, to cleave the β-lactam ring of the cephalosporin component with a concomitant ultimate release of the oxazolidinone. The released oxazolidinone can then cross the bacterial inner membrane and reach its intracellular ribosomal target, thereby killing the Gram-negative bacteria, as demonstrated using clinical isolates of the cephalosporinase-producing, highly troublesome pathogen *Acinetobacter baumannii*, and other Gram-negative bacteria, such as *Escherichia coli* DC0 and *Pseudomonas aeruginosa* KW799. An ingenious way to convert Gram-positive into Gram-negative effective antibiotics in the fight against multidrug resistant bacteria!

## 7. Cannabinoid-Induced Cell Death in Endometrial Cancer Cells: Involvement of TRPV1 Receptors in Apoptosis

Highlighted by Rafik Karaman

For centuries, cannabis has been utilized for tackling symptoms of a variety of diseases. For instance, cannabis has been used for the treatment of cancer-related symptoms, like pain and nausea. Cannabidiol (CBD), Δ^9^-tetrahydrocannabinol (THC), anandamide (AEA), endocannabinoids (eCBs), and 2-arachidonoylglycerol (2-AG) are considered as promising agents for the treatment of cancer patients. They have been shown to inhibit *in vitro* growth of tumor cells and induce tumor cell death. Recently, in a study by Fonseca et al. on endometrial cancer (cancer of the uterus), it was demonstrated that CBD is quite effective in causing death to many of the cancerous cells tested in the lab, whereas no significant effect was observed with THC. Unraveling the mechanism of this finding revealed that CBD was killing cancer cells by activating TRPV1 receptors, and the reduction in cell viability was caused by the activation of the apoptotic pathway. TRPV1 receptors are known to be one of CBD’s targets, and are not activated by THC. Thus, it was concluded that CBD is a promising medication for those patients who are not responsive to the traditional treatments [7]. 

## 8. Fenarimols, New Drug Candidates for a Great Social Problem

Highlighted by Carlo Siciliano

Eumycetoma is a neglected but grave infectious disease. Its transmission is favored by both geographical and economic factors, especially among resource-poor populations living in rural and urban areas of tropical and subtropical countries. Blindness, disability, deformities, impairment of childhood growth and development, and adverse pregnancy outcomes affect infected individuals. The clinical treatment of this disease has a limited success rate and requires a combination of surgery and prolonged pharmacological therapy. Conazoles have been the mainstay of medical treatment for decades. However, the etiological agents remain viable after treatment with these potentially fatal toxic drugs, which are usually too expensive for use in endemic regions.

A large class of fenarimol analogues has been subjected to *in silico*, *in vitro*, and *in vivo* screenings, for their activity against the causative agents of mycetoma diseases [8]. Four analogues were identified as the most efficient members of this class. A trifluoromethylaryl analogue of fenarimol showed the highest therapeutic efficacy, due to its minimal inhibitory concentration, lack of toxicity, and significantly reduced treatment time. Today, this novel drug candidate might represent a renewed and concrete hope to fast track and boost the discovery of cheaper therapies against socially important, but neglected, tropical diseases.

## 9. Late-Stage Lead Diversification: Metabolism Giving a Helping Hand at the Nanomole Scale

Highlighted by Maria Emília de Sousa

Late-stage diversification has gained considerable attention in drug discovery. This strategy offers the possibility of the rapid exploration of structure activity relationships (SAR), the preparation of biological probes, the generation of oxidized metabolites, and the blocking of metabolic hot spots, minimizing the risk for drug-drug interactions. Obach et al. [9] from Pfizer described a case-study of phosphodiesterase-2 inhibitors in which late-stage lead diversifications were accomplished by drug metabolism biosynthesis using P450 enzymes and liver microsomes. The breakthrough in this strategy was the use of quantitative cryomicroprobe NMR spectroscopy that allowed the gathering of spectral information at a submicromole scale. The authors reported how nanomole quantities of multiple products can be prepared simultaneously, screened, scaled-up, products isolated, and analyzed by quantitative NMR spectroscopy within a few days. One can imagine the investigation of alternative (bio)transformations which could be undertaken by applying this strategy.

## 10. Controlling Insatiable Appetite with a Melanocortin-4 Receptor Agonist in Patients with Leptin Receptor Defect

Highlighted by Katalin Prokai-Tatrai 

Leptin receptor mutations result in disruption of the satiety center within the brain, leading to insatiable appetite, and consequently, severe obesity. This condition cannot be treated permanently by weight loss surgery, highlighting the need for safe and efficacious pharmacological interventions. In a recent pilot study [10], it has been shown that individuals with specific leptin receptor deficiency responded well to the melanocortin-4 receptor (MC4R) agonist setmelanotide. MC4R is essential for transducing the satiety signal to the body. Setmelanotide is a peptide with the sequence acetyl-l-arginyl-cyclo[l-cysteinyl-d-alanyl-l-histidinyl-d-phenylalanyl-l-arginyl-l-tryptophanyl-l-cysteinyl]-amide, which was injected subcutaneously once per day to the subjects. Treatments with this peptide resulted in substantial and sustained loss of appetite and body weight without significant side effects, such as cardiovascular liability. The researchers have also discovered a new mechanism of action for setmelanotide that may explain its efficacy over other MC4R agonists. Altogether, the findings in this paper raise hope to counter genetic obesity in individuals suffering from deficiency in the hypothalamic leptin-melanocortin pathway. 

## 11. Phosphate Prodrug Strategy Is Applicable in Colon Drug Delivery

Highlighted by Jarkko Rautio 

Phosphate prodrug strategy where a phosphate group is either directly attached, or through a short linker such as OCH_2_ to the parent drug, has been very successful in improving dissolution-limited and/or solubility-limited oral absorption. In this paper [11], the design of phosphonooxymethyl prodrug was explored for the anti-HIV-1 agent, temsavir, which is categorized as a biopharmaceutics classification system (BCS) class II drug (low solubility and high permeability). The prodrug showed significantly higher aqueous solubility (>11 mg/mL at pH 1.5–8.2) than temsavir (~20 µg/mL at pH 2–9), with high oral bioavailability (80–122%) of the released temsavir after administration of the prodrug to preclinical species. However, dosing of the prodrug to humans revealed a short plasma half-life of 1.5 h for temsavir, which was different from studies in animals. That necessitated the development of an extended-release dosage form in order to achieve longer plasma exposure. Subsequently, prodrug release in the ascending colon afforded an improved pharmacokinetic profile of temsavir in humans. Therefore, this study showed for the first time that the expression of alkaline phosphatase in the lower GI tract is adequate to ensure effective phosphate prodrug conversion. The phosphonooxymethyl prodrug of temsavir, fostemsavir, has completed phase 3 studies. 

## 12. EphA2 Receptor Is a Key Player in the Metastatic Onset of Ewing Sarcoma

Highlighted by Catherine Guillou

Researchers of the Bellvitge Biomedical Research Institute (IDIBELL) have identified a potential new therapeutic target for Ewing sarcoma, the second most frequent bone cancer in children and adolescents, and a tumor known for its aggressiveness and tendency to metastasize. They correlate the EphA2 membrane receptor with the metastatic capacity of tumors in Ewing sarcoma. Researchers are currently working on nanoengineering a molecule capable of blocking EphA2 and delivering drugs in a targeted manner [12]. 

## 13. Discovery of Sulfonylfluoride Peptidomimetics as Targeted Covalent Inhibitors of Prolyl Oligopeptidase 

Highlighted by Michael Gütschow

Prolyl oligopeptidase (POP), a neuronal serine protease, cleaves post-proline bonds of small peptides. POP has emerged as a promising target for the development of enzyme inhibitors, which have already been shown to accelerate the clearance of aggregated α-synuclein, and are potentially useful for the treatment of cognitive and neurodegenerative disorders.

Ernest Giralt’s group at the Barcelona Institute of Science and Technology, together with Rob M.J. Liskamp’s group at the Utrecht Institute for Pharmaceutical Sciences, and further collaborators, have performed a structure-based design to develop novel shape-complementary POP inhibitors by assembling a peptidomimetic backbone of proline and 4-substituted proline as P1 and P2, tailored hydrophobic moieties as P3 groups, and a new sulfonyl fluoride electrophilic warhead (synthesized with difluoro(morpholino)sulfonium tetrafluoroborate) [13]. Several representatives of this chemotype possessed single-digit nanomolar IC_50_ values and high membrane permeability (as determined in PAMPA and MDCK assays). For a hit compound (*k*_inac_/*K*_i_ = 2 × 10^6^ M^−1^s^−1^), irreversible reaction with the active-site serine, more than 1000-fold selectivity for POP over two closely related proteases (DPPIV and FAP) and low-nanomolar activity in human cells was demonstrated. Thus, novel and highly potent POP inhibitors were introduced, whose selectivity ensures low cross-reactivity and whose membrane permeability grants access to the CNS.

## 14. Nanobiotics: A Bioinspired Approach to Fight Antibacterial Resistance

Highlighted by Stefania Galdiero

Lei et al. [14] have envisioned a strategy to induce the self-assembling of an antimicrobial peptide (AMP) in nanoparticle antibiotics (which they call nanobiotics) with excellent pharmacological properties. The C-terminal myristoylated human α-defensin 5 (HD5) assembled nanobiotic shows improved broad spectrum bactericidal activity *in vitro* and excellent tolerability *in vivo*. Furthermore, they found that the nano-compound was able to protect mice against skin infections by MRSA (methicillin resistant *Staphylococcus aureus*) and rescue them from *Escherichia coli* induced sepsis. This strategy provides an opportunity for further developments in next generation antibiotics to combat resistance which may be applied to the many natural AMPs. The authors present an innovative and fascinating approach to design nano-structures for extensive biomedical applications against a broad range of bacterial infections. A pioneering paper suggesting that the fight against antibacterial resistance is still open!

## 15. Enrichment-triggered Prodrug Activation: A New Concept for Targeted-releasing Prodrug Design

Highlighted by Hong Liu

Controlled activation of prodrugs precisely in target sites is strongly needed in targeted therapy. Creatively, by taking advantage of click reaction kinetics, Wang et al. [15] established a concentration-sensitive platform approach for bioorthogonal prodrug activation. The authors designed two different “click and release” systems to demonstrate the principle of the approach, using targeting tethers for prodrug enrichment and bioorthogonal click chemistry for prodrug activation. In both cases, the active drugs were released inside the mitochondrial matrix as a result of the enrichment-triggered click reactions. Moreover, mitochondria-targeted delivery provided remarkable augmentation of functional biological and therapeutic effects both *in vitro* and *in vivo,* compared with controls that did not result in enrichment. Therefore, this method provided a platform for targeted prodrug design that is amenable to conjugation with various molecules, and is not limited to cell-surface delivery. Collectively, this work demonstrated a new concept of enrichment-triggered prodrug activation and its critical feasibility of treating clinically relevant diseases, which may thus pave a new path for the development of targeted-releasing prodrugs for clinical use.

## 16. Following the Assembly of the Hepatitis B Virus Capsid in Real Time by Mass-Spectrometry

Highlighted by Luigi A. Agrofoglio

The hepatitis B virus capsid (HBV) is a promising therapeutic target, since its assembly is crucial to the completion of the viral life cycle. Using a charge detection mass spectrometry, researchers from Indiana University [16] have tracked in real time the assembly of the icosahedral capsid. This approach was able to investigate early intermediates in HBV capsid assembly as well as intact capsids, and data support pathways that are (or are not) susceptible to trapping. A facile pathway from dimer to the overgrown capsid occurs on an energy landscape that must be efficiently downhill. Salt concentration can modify the assembly reaction; under higher salt conditions, around half of the products of the initial assembly reaction have masses close to the *T* = 4 capsid, and the other half are stalled intermediates which emerge abruptly at around 90 dimers. When incubated at room temperature, the 90-dimer intermediates accumulate dimers, shift to higher mass, and merge with the capsid peak. These data have a potential to help in designing small molecules that block the capsid assembly.

## 17. Unlocking Promising Avenues to Identify 'Novel' Therapeutic Target Proteins and Candidate Drugs 

Highlighted by Jean-Marc Sabatier

Blood plasma proteins remain poorly studied although they play a central role in many key biological processes. The work by Sun et al. [17] gives a unique and more complete overview of the genetic architecture of the human plasma proteome in healthy blood donors, with the identification of almost 2000 genetic associations with ca. 1500 proteins (about 10% were so far identified). Interestingly, the authors were able to 'link' some specific genetic variations to particular regions that were reported to be associated with human diseases. This study contributed to a better understanding of the inter-dependency between specific genetic variations, human diseases, and levels of individual plasma proteins, thereby unlocking promising avenues for identifying 'novel' therapeutic target proteins, candidate drugs (even known drugs applied to other diseases), and further highlighting the potential risks of their use in humans. 

## 18. MK-7622: A First-in-Class M_1_ Positive Allosteric Modulator Development Candidate

Highlighted by Christopher Hulme

Despite continued set-backs with regulatory approvals of BACE inhibitors, first-in-class molecules designed to mitigate AD continue to move along the value chain. MK-7622 and its progression into Phase 2 trials is a timely success in lieu of the recent Merck BACE inhibitor failure in Phase 3. Beshore et al. [18] describe lead optimization efforts to address physicochemical property and safety issues, resulting in the discovery of the clinical candidate MK-7622, a very selective positive allosteric modulator of the M_1_ receptor. Interestingly, the pharmaceutical industry has pursued the discovery of selectively activating M_1_ modulators for decades, and the publication of this work with an associated structure should spur further efforts in the field to improve cognition in Alzheimer’s patients. 

## 19. A Novel Class of Docosahexaenoyl Ethanolamide (DHEA) Epoxides that Exhibit Anti-Inflammatory and Anti-Tumorigenic Properties

Highlighted by George Kokotos

Dietary omega-3 fatty acids such as eicosapentaenoic acid (EPA) and docosahexaenoic acid (DHA) have been shown to suppress tumor growth and progression by their conversion to anti-inflammatory and anti-tumorigenic lipids. DHA and EPA can be converted by the *N*-acyl ethanolamine synthesis pathway to endocannabinoids docosahexaenoyl ethanolamide (DHEA) and eicosapentaenoyl ethanolamide (EPEA). Recently, the endogenous production of a previously unknown class of ω-3 PUFA-derived lipid metabolites that originates from the crosstalk between endocannabinoid and cytochrome P450 epoxygenase metabolic pathways was reported [19]. In neuroinflammation studies, 17,18-epoxyeicosatetraenoic acid-ethanolamide (EEQ-EA) and 19,20-epoxydocosapentaenoic acid-ethanolamide (EDP-EA) dose-dependently abated proinflammatory IL-6 cytokines while increasing anti-inflammatory IL-10 cytokines, in part through cannabinoid receptor-2 activation. More recently, EDP-EAs have been shown to exhibit anti-angiogenic, anti-tumorigenic, and anti-migratory properties in osteosarcoma [20]. EDP-EAs were found to be increased by ~80% in metastatic lungs versus normal mouse lungs, while significant differences in the apoptotic and anti-migratory potency of the different EDP-EA regioisomers were observed. These naturally occurring molecules may be of therapeutic significance to prevent metastasis of tumors.

## 20. Click Chemistry-based Discovery of Orally Active Hypoxia Inducing Factor Prolyl Hydroxylase Inhibitors with Favorable Safety Profiles for the Treatment of Anemia

Highlighted by Qidong You

Anemia is a frequent complication of chronic kidney disease (CKD), because failing kidneys produce insufficient erythropoietin (EPO) to maintain normal red blood cell levels. Currently, intravenous administration of recombinant human EPO (rhEPO) is the standard treatment to ameliorate anemia. However, its serious side effects and the requirement for hospitalization deter many patients. Today, inhibiting hypoxia-inducing factor prolyl hydroxylase 2 (HIF-PHD2) by an orally active small molecule is regarded as an effective strategy to stabilize HIF-α and then improve endogenous EPO level for anemia treatment. Recently, Wu et al. [21] reported the details of a study to screen, optimize, and identify triazole compounds as potent HIF-PHD2 inhibitors based on click chemistry. Of particular note was the orally active HIF-PHD2 inhibitor *N*-(5-(1-(3-(4-chlorophenyl)propyl)-1*H*-1,2,3-triazol-4-yl)-3-hydroxypicolinoyl)glycine (IC_50_ = 62 nM), which was almost ten times more active than the phase III drug FG-4592 (IC_50_ = 591 nM). Furthermore, it can upregulate the hemoglobin of anemic mice (120 g/L) to normal levels (160 g/L), with no apparent toxicity observed *in vivo*. These results confirm that it is a promising candidate for the treatment of renal anemia.

## 21. Acyclovir as an Ionic Liquid Cation or Anion Can Improve Aqueous Solubility

Highlighted by Paula A. C. Gomes 

Ionic Liquids (ILs) have unique properties which are appealing for diverse applications, from eco-friendly solvents (1st generation ILs) to tunable materials (2nd generation ILs). A decade ago, the 3rd generation of ILs emerged based on use of active pharmaceutical ingredients (APIs) to produce bioactive ILs (API-ILs) that may transform medicinal chemistry and the pharmaceutical industry [22]. Exciting findings have been reported, including antimicrobial API-ILs which are active even against antibiotic-resistant bacteria [23]. Amongst the many benefits of API-ILs as drugs, their water-solubility and tunability are the greatest, and have been addressed in a recent original article by Shamshina et al. [24]. In this work, the solubility of the anti-retroviral drug acyclovir in different media was tuned by the proper choice of the drug’s ionization state (cation or anion) and counter-ion. This emblematic example highlights the urgent need to regulate pharmaceutical development of API-ILs. After all, a paradigm shift has to happen before running out of options to tackle the rise of one of the major health threats of our times, antibiotic resistance.
